# Implementation quality of whole-school mental health promotion and students’ academic performance

**DOI:** 10.1111/j.1475-3588.2011.00608.x

**Published:** 2012-02

**Authors:** Katherine L Dix, Phillip T Slee, Michael J Lawson, John P Keeves

**Affiliations:** Flinders Centre for Student Wellbeing and the Prevention of Violence, School of Education, Flinders UniversityAdelaide, South Australia 5001. E-mail: katherine.dix@flinders.edu.au

**Keywords:** Mental health, academic performance, intervention quality, primary school children, social-emotional competencies

## Abstract

**Background:**

This paper argues for giving explicit attention to the quality of implementation of school-wide mental health promotions and examines the impact of implementation quality on academic performance in a major Australian mental health initiative.

**Method:**

Hierarchical linear modelling was used to investigate change in standardised academic performance across the 2-year implementation of a mental health initiative in 96 Australian primary (or elementary) schools that was focused on improving student social-emotional competencies.

**Results:**

After controlling for differences in socioeconomic background, a significant positive relationship existed between quality of implementation and academic performance. The difference between students in high- and low-implementing schools was equivalent to a difference in academic performance of up to 6 months of schooling.

**Key Practitioner Message**

- Given the known relationship between student academic achievement and mental health, many nations are mounting school-based mental health interventions: however, the quality of program implementation remains a concern
- The Australian KidsMatter primary school mental health intervention enabled the development of an Implementation Index allowing schools to be grouped into low- to high- implementing schools
- The quality of implementation of KidsMatter appears to be positively associated with the level of student academic achievement, equivalent to 6 months more schooling by Year 7, over and above any influence of socioeconomic background

## Introduction

The improvement in schools’ abilities to enhance student outcomes continues to be a central focus of governments in Australia (Angus, Olney, & Ainley, 2007; Gillard, 2010) and internationally (Ainscow & West, 2006; Fullan, 2007; Resnick, 2010; Sammons, 2007). Among the many priorities also identified as important in schools, the development of students’ mental health and wellbeing is considered integral (Elias et al., 1997; WHO, 2005). Related research indicates that ‘Schools will be most successful in their educational mission when they integrate efforts to promote children’s academic, social, and emotional learning’ (Zins et al., 2004, p.3), and that ‘strong bonds between student behaviour, attainment and learning and their social and emotional development’ are central (Sammons, 2007, p.37). The level of interest of governments is shown by the recent development of nation-wide initiatives to address students’ mental health and wellbeing (e.g. DCSF, 2009; CASEL, 2008; ENSEC, 2009; KidsMatter, 2009).

However, despite the emergence of a large number of school-based programs that foster positive mental health, there is growing concern about the effective implementation of such programs (Adelman & Taylor, 2000; Domitrovich & Greenberg, 2000; Durlak & DuPre, 2008; Greenberg, 2004). Moreover, Domitrovich and Greenberg (2000) raised concerns regarding the lack of studies reporting the relationship between the quality of implementation of mental health promotion initiatives and student outcomes.

In response to these concerns, Slee et al. (2009) developed a measure of implementation quality that was positively associated with participants’ views of how well a school-wide mental health promotion program met the needs of the students involved in the program. The program being implemented was the Australian KidsMatter Primary^1^ intervention. Importantly, the relationships examined by Slee et al. (2009) did not consider the impact of social-emotional competencies upon students’ academic outcomes, nor did it control for the socioeconomic context of the school, issues raised elsewhere (Angus et al., 2007). Accordingly, a major focus in the current study is the influence of the quality of implementation of KidsMatter on student academic outcomes. In this paper we examine the relationship between students’ academic performance and the implementation quality of the KidsMatter intervention program, after controlling for the effects of the socioeconomic background of the students’ families. For this purpose, we bring together two large Australian datasets, namely the KidsMatter Primary Evaluation data (Dix et al., 2010) and the National Assessment Program - Literacy and Numeracy (NAPLAN) achievement data (ACARA, 2008)^2^.

### KidsMatter Primary

KidsMatter Primary is an Australian mental health promotion, prevention and early intervention initiative that was trialled in 100 schools. The implementation of the KidsMatter trial involved a whole-school systemic approach that was guided by a conceptual framework, an implementation process, and provision of additional resources (Graetz et al., 2008). The intervention was designed to support and involve all members of the school community, including school leadership, teachers, parents and students, with the aims of (a) improving mental health and wellbeing of the students, (b) reducing mental health problems among students, and (c) achieving greater support for students experiencing mental health problems. It did so through a program derived from a four-component conceptual framework focusing on (1) positive school community, (2) social and emotional learning for students, (3) parenting support and education, and (4) early intervention for students experiencing mental health difficulties. The evaluation of the KidsMatter trial is reported in Slee et al. (2009).

### Socioeconomic background, mental health and academic achievement

Research has shown that socioeconomic status (SES) is associated with a wide range of health, cognitive, educational, and socio-emotional outcomes in children (Bradley & Corwyn, 2002). At the level of general health, research over past decades has shown a consistent relationship between health status and SES, such that ‘across the full range of SES, higher SES is associated with better health’ (Adler & Snibbe, 2003, p. 119). For child and adolescent mental health, socioeconomic deprivation is recognised in many societies as one of the key risk factors (Patel et al., 2008). As argued by Kazdin (1993) almost two decades ago, undertaking any examination into the effects of an intervention on mental health should account for the impact of differences in socioeconomic background. Similarly, in an extensive meta-analysis examining the relationship between SES and academic achievement, Sirin (2005, p.447) concluded ‘researchers must assess the student’s family background regardless of their main research focus’. Accordingly, the availability of a set of national SES data developed to compare academic performance in Australian schools (ACARA, 2010), including schools in the KidsMatter trial, made possible the inclusion of socioeconomic background in the current analysis.

### Social-emotional wellbeing and academic achievement

The relationship between social-emotional competencies and academic achievement has been under investigation for over 40 years (Purkey, 1970). One early study was that of Davison and Greenberg (1967) who investigated children’s multiple views of self, including personal- and social-competence, and found positive relationships of these with academic achievement. More recently, a meta-analysis of over 200 school-based studies on the impact of universal interventions to enhance students’ social-emotional learning found benefits in school achievement, among others (CASEL, 2007, 2008). Gains produced by school-based programs translated into an 11 percentile point improvement in achievement test scores. These findings were extended in a further review to include gains of up to 17 percentile points (Payton et al., 2008).

This and other research suggest that well-planned and well-implemented opportunities for supporting the social-emotional development of students can positively affect academic outcomes (Greenberg et al., 2003; Gumora & Arsenio, 2002; Malecki & Elliott, 2002; Teo et al., 1996; Welsh et al., 2001; Wentzel, 1993; Wood, 2006; Zins et al., 2004). In the context of the KidsMatter initiative, evidence related to the impact of the initiative on students’ academic outcomes was indirect, since the main aim of KidsMatter was to improve students’ mental health and wellbeing, and not learning outcomes. Nevertheless, Slee et al. (2009) reported that 92% of teachers across 100 schools strongly agreed, that ‘Students who are socially and emotionally competent learn more at school’. Across the 2-year study these stable and strong beliefs about the benefits to learning of well-developed social-emotional competencies were also evident in interview data gathered from KidsMatter school principals (Slee et al., 2009, p.74). This anecdotal evidence precipitated our further investigation of the relationship between the quality of implementation of KidsMatter and academic achievement, using more direct evidence on academic performance.

## Method

### Participants

Of the 260 primary (elementary) schools volunteering to participate in KidsMatter, 100 schools were selected to achieve representation across Australian states, location (metro, rural, remote), size, and sector (Catholic, independent, public). The participants comprised a random stratified sample of up to 76 students in each of the 100 schools, giving preference in selection to students aged 10 years, who were the focus of the KidsMatter intervention. The resulting participants at the first measurement occasion (Time 1) comprised parents and teachers of 4980 students (70% response rate). Students’ average age was 9.6 years (*SD* = 1.6) and 48% were male. For the 1393 teacher participants, the average level of teaching experience was 15.1 years (*SD* = 10.8) and 85% were female. The family context reported by parents in the sample comprise 83% two-parent families and 17% single-parent families, comparable to the national profile (Australian Institute of Family Studies, 2011).

### Procedure

During the 2 years of the KidsMatter trial (2007/08), questionnaires on a variety of aspects of school and student functioning were administered to the students’ teachers on four occasions and to their parents on three occasions. In brief, the questionnaires sought information on areas of school engagement and implementation of the initiative, impact on the school in general, impact on teachers and families, and impact on student social-emotional competence and on their mental health. In addition, data were available in reports received from school leadership at the end of the trial and in regular progress reports related to each site provided throughout the trial by eight state-based KidsMatter project officers. Further details of questionnaire items, sampling procedure, and data collection procedures are provided in Slee et al. (2009) and Dix et al. (2010).

### Ethics

Ethics applications were submitted and approvals received from Flinders University, and also from over 30 school, jurisdiction and departmental bodies in all Australian States and Territories, in addition to consent from principals of the 100 participating schools.

### Measures

#### The KidsMatter Implementation Index

Given the complexity of implementing a mental health and wellbeing initiative school-wide, it was anticipated that some schools would engage more readily than others with the four KidsMatter components, implementation process, professional learning and resources, and so would be better able to effect change. In response to concerns about evaluating the quality of implementation (Durlak & DuPre, 2008), an Implementation Index was developed, as reported in Slee et al. (2009), using Latent Class Analysis (Muthén & Muthén, 2007). Although the theoretical development of the Implementation Index has been presented elsewhere (Dix et al., 2010), some background is provided here to inform the analysis in this paper. To identify schools as being high or low implementers of KidsMatter, a framework derived from the work of Domitrovich et al. (2008) was used to develop an Implementation Index that represented the quality of schools’ implementation of KidsMatter in terms of three elements: fidelity of implementation, extent of the dosage delivered, and the quality of the delivery process. The Implementation Index was informed by data derived from the views of those experiencing the KidsMatter intervention (parents and teachers), as well as those providing dedicated support for the implementation (KidsMatter project officers). Information used in the formation of the Index was generated from measures available on the last of the four assessment occasions of the evaluation, at a time when it was considered that a reasonable level of implementation would have been achieved.

The resulting Implementation Index was found to be effective in differentiating between high- and low-implementing schools, with respect to the development of social-emotional competencies (Slee et al., 2009). Differences between high- and low-implementing schools were apparent in two main areas. The first was adherence to the steps prescribed for program implementation (e.g. defining issues, setting goals, evaluating strategies, and reviewing and adjusting plans). The second area identified differences in levels of involvement of parents and teachers, such as active involvement of the school leadership team and of the whole staff in planning, and the degree of encouragement of parental involvement.

#### Measure of socioeconomic background

For the purposes of this analysis, the Index of Community Socio-Educational Advantage (ICSEA) was selected as an adequate measure of social disadvantage, notwithstanding some criticism of this measure (Preston, 2010). ICSEA is an index of the socioeconomic background of the students at the school, with more advantaged schools having a higher ICSEA and schools with students from more disadvantaged backgrounds having a lower ICSEA. The ICSEA Index, based on census data, was developed specifically for the Australian *My School* website (ACARA, 2010) to enable meaningful comparisons of standardised achievement scores between schools across Australia. Although ICSEA was designed as a measure to predict academic performance, our preliminary correlation analysis further supported the inclusion of ICSEA in our model, with medium (0.42, *p* < .05) and small (0.24, *p* < .05) correlations associated with academic performance and with the KidsMatter Implementation Index respectively.

#### Measure of student academic performance

Given that KidsMatter was interested in, but was not focused specifically on, improving student academic outcomes, no detailed assessment of academic performance was conducted within the evaluation of the KidsMatter trial. However, one item pertaining to academic outcomes was asked of teachers on four occasions for each student participating in the evaluation, namely, ‘KidsMatter has led to improvements in this student’s schoolwork’. This longitudinal data provided an opportunity to examine change in ratings for students over time. Over the 2 years, 14% more teachers strongly agreed (scored 6 or 7) that KidsMatter had led to improvements in students’ schoolwork, equivalent to a medium^3^ (*β* = 0.30, *p* < .05) effect size. While it suggests that there were perceived improvements in academic performance due to the impact of KidsMatter, this one item, based on teacher perception alone, was recognised to be a limited and indirect indicator.

An alternative national standardised measure of academic performance data collected within the period of the KidsMatter trial was available. Slee et al. (2009, p.74) reported a response from one school principal, who was asked about whether KidsMatter had a role to play in terms of literacy and numeracy (LAN) results: ‘I believe that happy, healthy schools get good [LAN] results’. The ‘LAN results’ mentioned referred to the Australian National Assessment Program - Literacy and Numeracy (NAPLAN). These data were chosen due to their availability for all schools in the KidsMatter trial, their comparability across schools, and their timeliness (ACARA, 2008; Masters et al., 2008).

Along with the ICSEA rank, the mean achievement scores for each cohort in Years or Grades 3, 5 and 7 during 2008 were collected from the *My School* website for each KidsMatter school. These Year or Grade levels were considered most likely to include the students who were involved in KidsMatter during the evaluation trial in 2007–08. The resulting database consisted of average student achievement for Years 3, 5 and 7 for each KidsMatter school in the five key areas of Reading, Writing, Spelling, Grammar-Punctuation, and Numeracy. However, due to differences between Australian state education authorities, Year 6 was the final year of primary schooling in some states, and so data for Year 7 were not available for approximately half the schools. It was also found that four schools did not have sufficient data and these were removed from the subsequent analysis (final school *n* = 96). All achievement data derived from the information on the *My School* website^4^ was publicly available and did not involve obtaining access to confidential records.

**Figure 1:**
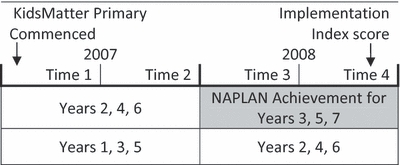
Students participating in KidsMatter and academic achievement tests

The broad relationship between the students involved in KidsMatter during 2007–08, and their completion of the literacy and numeracy tests during 2008, is presented in Figure 1. It should be noted that while KidsMatter was designed to be implemented as a whole-school approach, the parents and teachers of Year 5 students were the target sample. Given that academic achievement data were only reported as an average at each Grade for Years 3, 5 and 7, it is not known precisely how intact the student groups had remained. Estimates from the KidsMatter data suggested that there was a transitory population of approximately 5% of students who moved between schools (and therefore in and out of KidsMatter schools). For the purposes of this analysis, it was assumed that the academic achievement scores for students in Years 3, 5 and 7 represented the majority of students who were involved in KidsMatter, and of their parents and teachers who participated in the evaluation and contributed to the ratings on the Implementation Index. These qualifications need to be borne in mind in interpreting the findings from the present analysis. Figure 1 also shows the NAPLAN assessment time in relation to the timing of data used for the KidsMatter Implementation Index, assessed at the end (Time 4) of the 2-year evaluation in 2008.

### Statistical analysis

A two-level hierarchical linear model, using HLM-5 (Raudenbush et al., 2000), was employed to examine the relationships between school-level characteristics and student-level academic outcomes (Raudenbush & Willms, 1995). This regression technique provides a way of examining whether the effects of school factors vary among communities by taking into account the hierarchical structure of educational data (Bryk & Raudenbush, 1992). A reduction in deviance from the null model was used to indicate an improved model fit and *p* < .05 was used to signify acceptable statistical significance (Bryk & Raudenbush, 1992). The purpose of testing the regression model was to address the broad question of whether involvement in KidsMatter was associated with improvement in student academic performance.

## Results

An academic achievement score for each Year level in each of the 94 KidsMatter schools was calculated as the average of the five NAPLAN measures of Reading, Writing, Spelling, Grammar-Punctuation, and Numeracy (*M* = 450.6, *SD* = 62.0). For the purposes of this investigation, creating a single score by combining the five learning areas was useful and was supported by significant inter-item correlations, *r* > .93, α = .99 (Field, 2009; Tabachnick & Fidell, 2007).

**Figure 2:**
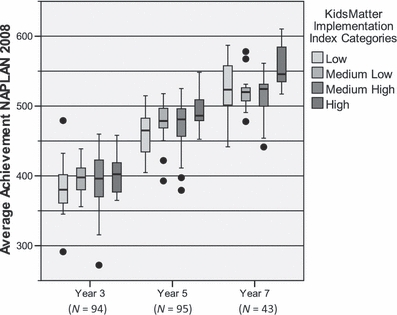
Distribution of Years 3, 5 and 7 NAPLAN achievement scores in 2008 according to level of KidsMatter Implementation quality

For the purposes of visual inspection, NAPLAN scores were grouped according to Low, Medium-low, Medium-high and High implementing schools, based on Implementation Index cut-points at the mean and plus or minus one standard deviation (*M* = 159.7, *SD* = 26.9). The box-plot in Figure 2 shows the distinct differences in levels of achievement of students in Years 3, 5 and 7, as expected, but also suggests trends in the pattern of relationship between the level of achievement and level of implementation quality.

**Figure 3:**
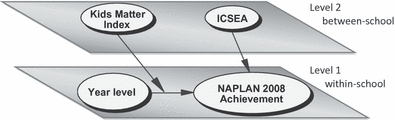
Theoretical two-level HLM model of factors influencing academic achievement

The basic relationship presented in Figure 2 was modelled using two-level HLM analysis. Figure 3 presents the theoretical regression model with Year (Grade) as a Level 1 predictor of NAPLAN achievement scores, and the Implementation Index and socioeconomic ICSEA background as Level 2 predictors. With a reduction in deviance of 461.70 and an additional two degrees of freedom, Table 1 presents the regression coefficients for the final two-level HLM model for academic achievement.

**Table 1:** Final estimations of the two-level HLM model for academic achievement

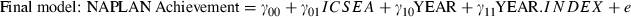				

Fixed effect	Coefficient	Std error	*t*-ratio	*p*-value
INTERCEPT, *γ*_*00*_	296.00	4.63	63.94	.00
ICSEA, γ_01_	0.31	0.02	12.87	.00
YEAR, γ_10_	34.34	0.82	41.64	.00
YEAR.INDEX, γ_11_	0.83	0.33	2.48	.01

**Figure 4:**
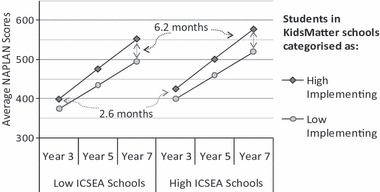
The relationship between low and high implementation of KidsMatter and achievement

Figure 4 presents the cross-level interaction of KidsMatter Implementation Index with NAPLAN academic performance, controlling for ICSEA background. The ICSEA scores were developed by ACARA (2010) for the purpose of comparing schools of similar socioeconomic background. As expected, schools with higher ICSEA scores in general, achieved higher performance scores than schools with children from low socioeconomic backgrounds (*γ*_*01*_ = 0.31, *p* < .01). For every unit increase in ICSEA, achievement increases by 0.31 NAPLAN test score units. For schools 80 ICSEA units apart (equivalent to 1 *SD*), this could equate to a difference of 25 test units. Interestingly, although the moderating effect of Index was significant at the .01 level, an alternative model, which considered the indirect influence of ICSEA moderating the effect of Year level, was not significant.

As expected, the slopes of each line, as a function of Year level, were found to be highly significant, showing large differences in achievement between each Grade (*γ*_*10*_ = 34.34, *p* < .01). This effect of Year level can be interpreted as indicating that one year could be considered equivalent to 34 test units. This interpretation enables the generation of a calibration scale.

Of particular interest was the relationship between KidsMatter Implementation and Year level, indicated by arrows in Figure 4 that show the vertical differences between line pairs. There were significant differences in standardised achievement scores between low- and high-implementing schools, equivalent to a small effect size of 0.26 (*p* < .05) (Cohen, 1992). Moreover, the cross-level interaction of Implementation Index with Year level (*γ*_*11*_ = 0.83, *p <* .01), suggested that KidsMatter was more effective for older students who were in the higher Grades. Given that the target sample for the evaluation was students aged 10 years, it was likely that they and their teachers were more involved with KidsMatter during the 2-year trial. In addition, when the Index was tested as a direct influence on achievement it was found to be non-significant, further suggesting that a *progressive* change in schools across the Year levels was occurring. This progressive difference across Year levels would seem to be associated with the quality of implementation of KidsMatter.

Making use of the calibration scale, a difference in academic performance between high- and low-implementing schools based on three standard deviations, was calculated for Years 3, 5 and 7, using the following formula:


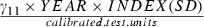


Difference in academic achievement at:


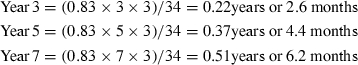


It can be estimated that at the Year 3 level in the high-implementing schools, compared with the low-implementing schools, students were 2.6 months ahead in academic achievement, rising to 4.4 months ahead at Year 5, and to 6.2 months ahead by Year 7. This analysis suggests that the difference in academic achievement for students in high- and low-implementing schools could be as much as 6 months of schooling, after controlling for socioeconomic background. It should also be noted that the difference in performance of 2.6 months of school achievement at the Year 3 level suggests that the KidsMatter program had an influence during the junior primary years. Moreover, as a consequence of being in a high-implementing school, students can be estimated to have gained 3.6 months (6.2–2.6 months) in school learning of the basic skills of Literacy and Numeracy as assessed by the NAPLAN tests over the 4-year period between Years 3 and 7. These effects are illustrated in Figure 4 and are considered to be the same for the high and low ICSEA schools, as a consequence of the moderating effect of ICSEA on the relationship between Year and NAPLAN achievement.

## Conclusions

This paper makes use of hierarchical linear modelling to examine the relationship between the quality of implementation of an Australian school-wide mental health and wellbeing initiative and student academic performance. The findings provide evidence of practical significance and support the notion that schools that implemented KidsMatter well also had improved learning outcomes for students, equivalent to 6 months more schooling by Year 7, over and above any influence of socioeconomic background. The results of the two-level HLM analysis undertaken at the Year level cannot be taken to indicate unequivocally that KidsMatter was lifting student performance on numeracy and literacy. However, over the 2-year evaluation, a 14% shift in teachers’ views that *‘*KidsMatter has led to improvements in this student’s schoolwork’ gives further strength to the notion that academic improvement in students did occur. Schools that were committed long-term to the effective school-wide implementation of the KidsMatter mental health initiative may well have better positioned themselves to support both students’ mental health and academic outcomes. The words from one principal interviewed during the KidsMatter evaluation captured this important idea:

We found that happy kids and contented kids, and kids who know how to interact better with one another, are much better learners. So we see things going together very much hand in glove. (Slee et al., 2009, p.74)

We have referred earlier to limitations of the data available for this analysis. Academic performance data were not collected as part of the KidsMatter evaluation. However, the availability of standardised achievement data that were collected in the timeframe of KidsMatter provides a well-founded basis for the analyses carried out. Schools that implement initiatives well may in fact do many things well, or may have particular characteristics that this analysis has not accounted for, although the significant effect of the socioeconomic background of the schools was considered in the analysis. While KidsMatter was the main initiative being implemented in these schools, they may have had other programs concurrently in place to support academic performance. In order to strengthen the claim that the quality of implementation of mental health initiatives such as KidsMatter improves student social-emotional competencies and, in turn, academic performance, research is needed that brings together measures of social-emotional competency, implementation quality and standardised academic performance at the *student level.* These findings provide impetus for further examination of the quality of implementation as part of mental health initiatives, not just assessed at the end of the initiative, as occurred here, but as an integral and regular part of any intervention.

## Notes

1 For details of the KidsMatter initiative for Australian primary schools and its associated resources see http://www.kidsmatter.edu.au/primary/

2 The Australian Curriculum, Assessment and Reporting Authority (ACARA) has been established by the Australian Government as an independent authority responsible for national curriculum, assessment, data collection and reporting that supports 21st century learning for all Australian students. NAPLAN is managed by ACARA.

3 Effect size was based on a regression coefficient equivalent to a part correlation with 0.10, 0.24, and 0.37 as indicative of the cut points for, small, medium and large, respectively (Dix et al., 2010; Ferguson, 1971; Kirk, 1996).

4 There has been, and continues to be, debate regarding the nature of the achievement data derived in the manner that it is for the purposes of the Australian National assessment program but this debate is not the focus of the present study.
